# Design and Evaluation of a Digital Health App (SingaporeWALK) for Active Aging: Pre-Post Intervention Study

**DOI:** 10.2196/68937

**Published:** 2025-08-22

**Authors:** Huanyu Bao, Sowmiya Meena Siva Subramanian, Sai Ganesh Sarvotham Pai, Navrag B Singh, Kai Zhe Tan, Tan Phat Pham, Feihong Pan, Yin-Leng Theng, Edmund Lee

**Affiliations:** 1 Department of Media & Communication, City University of Hong Kong, M5010, 5/F Run Run Shaw Creative Media Centre, 18 Tat Hong Avenue, Hong Kong, China (Hong Kong), 852 34428868; 2 Wee Kim Wee School of Communication and Information, Nanyang Technological University, Singapore, Singapore, Singapore; 3 Singapore-ETH Centre, Future Health Technologies Programme, CREATE Campus, Singapore, Singapore, Singapore; 4 Institute of Digital Medicine, City University of Hong Kong, , Hong Kong, China (Hong Kong)

**Keywords:** mHealth, exergames, digital health technologies, wearables, digital divide, usability testing

## Abstract

**Background:**

The global trend toward population aging poses significant challenges for maintaining older adults’ health and well-being, particularly in multicultural urban environments like Singapore. Despite the potential of digital health interventions, older adults face substantial barriers to technology adoption, including complex interfaces and culturally inappropriate content. Existing mobile health apps often fail to integrate physical, nutritional, and mental health components or accommodate the needs of multicultural older adult populations.

**Objective:**

To address gaps in mobile app design for older adults and bridge the digital divide, this research aimed to create and evaluate the SingaporeWALK (SGWALK) app, a culturally inclusive digital health solution promoting active aging through community-based interventions among Singapore’s older adults.

**Methods:**

The SGWALK app was developed using participatory design methodology involving iterative testing with older adults to ensure appropriateness and usability. The app integrates 3 core components: exergames (Fruit Ninja, Piano Step, and Arctic Punch) aligned with Singapore’s exercise guidelines for older adults, nutrition tracking based on local dietary recommendations, and mental well-being assessment using the Mental Health Continuum–Short Form. Following development, a 4-week pre-post intervention study was conducted with 48 participants (aged 60‐85 y) randomly allocated to 4 conditions: conventional exercise, exergames only, exergames with health coach support, or exergames with peer support. In total, 5 wearable inertial measurement unit sensors captured movement data during weekly 30-minute supervised sessions at community centers. Primary outcomes included changes in physical activity metrics, technology acceptance, and mental well-being measured through pre- and postintervention assessments.

**Results:**

The 4-week intervention demonstrated significant improvements across multiple health domains. Physical activity measures showed a 6.5% increase in maximum acceleration (*t*_47_=3.82, *P*<.001), while nutritional tracking revealed steady improvements in healthy eating patterns throughout the intervention period. Mental health assessments indicated that participants classified as “mentally well” consistently outperformed the “moderate” group across physical activity measures. Technology acceptance showed substantial enhancement, with willingness to use health apps increasing from mean 3.18 (SD .79) to mean 3.95 (SD .82; *t*_29_=−3.63, *P*<.001), and perceived ease of use improving from mean 3.01 (SD .70) to mean 3.76 (SD .68; *t*_29_=−4.08, *P*<.001). Additionally, participants developed more efficient movement patterns over time and formed supportive social relationships during the community-based implementation.

**Conclusions:**

The SGWALK app shows promise for promoting active aging and reducing technology barriers among Singapore’s older adults. The community-based implementation model, bilingual interface, integrated health monitoring approach, and sensor-based movement tracking offer potential advantages over existing solutions. These findings provide useful insights for researchers and practitioners developing digital health interventions for older adult populations in multicultural urban settings.

## Introduction

### Background

Singapore’s demographic landscape is undergoing a profound transformation, with citizens aged 65 years and older projected to constitute nearly a quarter of the population by 2030 [1]. This rapid aging presents both opportunities and challenges, necessitating innovative approaches to safeguard the health and autonomy of older adults. The concept of active aging—a multidimensional framework encompassing physical activity, nutrition, and mental well-being—has gained prominence as a strategy to mitigate age-related decline [2]. Research indicates that regular physical activity in older adults can significantly reduce the risk of chronic diseases, improve mental health, and enhance cognitive function [3]. Studies have highlighted the critical role of physical exercise in preventing cardiovascular diseases and promoting musculoskeletal health, which is essential for maintaining mobility and independence in later life [4,5]. However, translating these evidence-based benefits into practice remains fraught with barriers, particularly in technology adoption among older adults.

Despite the promise of digital health solutions, a critical obstacle is the pervasive digital divide affecting older adults. In Singapore, many older adults report hesitancy toward health technologies, citing concerns about privacy, usability, or perceived irrelevance to their needs [6]. While mobile health (mHealth) applications show potential, such as exergaming platforms to enhance physical activity [7] or wearables for mobility monitoring [8], most fail to accommodate the unique requirements of older adult users. Common shortcomings include cognitively demanding interfaces, lack of multilingual support, and fragmented features that neglect the synergistic relationship between physical, nutritional, and psychological health [9]. This gap in design inclusivity risks excluding the very population these tools aim to empower.

To address these limitations, we introduce SingaporeWALK (SGWALK), a mobile application co-designed with older adults using participatory methods to promote active aging within community settings. SGWALK integrates 3 core functionalities: (a) physical activity promotion through exergames such as Fruit Ninja and Piano Step, aligned with Singapore’s national exercise guidelines for older adults; (b) nutrition tracking with goal-setting features focused on hydration, dairy intake, and balanced meals; and (c) mental well-being monitoring using validated psychological assessment tools. Accessibility is central to SGWALK’s design, with bilingual (English–Chinese) interfaces, enlarged visual elements, and seamless integration with inertial measurement unit (IMU) sensors for real-time movement analysis.

By anchoring the intervention in community centers—important social hubs for Singaporean seniors—this study contributes to the field of gerontechnology in several key ways. First, it offers a replicable framework for co-designing digital health interventions with older adults, highlighting the importance of cultural relevance and usability. Second, it demonstrates the practical integration of exergaming, sensor-based mobility tracking, and holistic health monitoring in real-world community environments. Third, it provides empirical insights into addressing adoption barriers among older users, with broader implications for multicultural, urban aging populations.

### Literature Review: Active Aging and Digital Health Interventions

The concept of active aging has evolved beyond mere physical health to encompass holistic well-being, including nutritional status and mental health [2]. Research demonstrates that technology-enabled interventions can effectively support these dimensions: physical activity apps reduce sedentary behavior in older adults [10], while nutrition-tracking tools improve dietary adherence in populations with diabetes [11,12]. However, adoption rates remain low among older adults, with studies attributing this to complex interfaces, privacy concerns [6], and lack of cultural adaptation [13]. In Singapore, where many older adults speak non-English languages at home, these barriers are exacerbated by linguistic mismatches in existing apps [6].

Among digital health interventions, exergaming has emerged as a particularly promising strategy to engage older adults in physical activity. Studies show Kinect-based games improve balance and upper-body mobility [7], while wearable sensors enable precise tracking of gait and fall risk [8]. However, most solutions focus on individual use, neglecting the social dynamics of community settings—a critical gap given that group activities increase adherence compared to solo interventions [10]. Similarly, nutrition apps for seniors often fail to account for age-related needs or local dietary habits [13], while mental health apps using validated questionnaires rarely integrate data with physical activity metrics [14].

To overcome these limitations, effective digital health tools for older adults must address several key design challenges. Accessibility remains paramount, with studies showing the importance of interfaces tailored to older adults’ specific needs [9]. Simplified navigation is equally critical, as multi-step workflows increase abandonment rates compared to single-task interfaces [15]. Motivation presents the third challenge, where personalized feedback boosts engagement more than generic prompts [16].

Despite these known requirements, current active aging technologies exhibit persistent limitations that this study addresses. First, many existing apps treat physical, nutritional, and mental health as isolated domains [13], contrary to established active aging frameworks. Second, while existing apps claim multilingual support, few adapt content to local contexts like Singapore’s food culture [6]. Third, most solutions prioritize individual use, despite evidence that group settings enhance adherence [10]. These gaps highlight the need for integrated, culturally adapted digital health solutions that can effectively engage older adults in community settings.

### SingaporeWALK App Development

#### Overview

**Figure 1.**
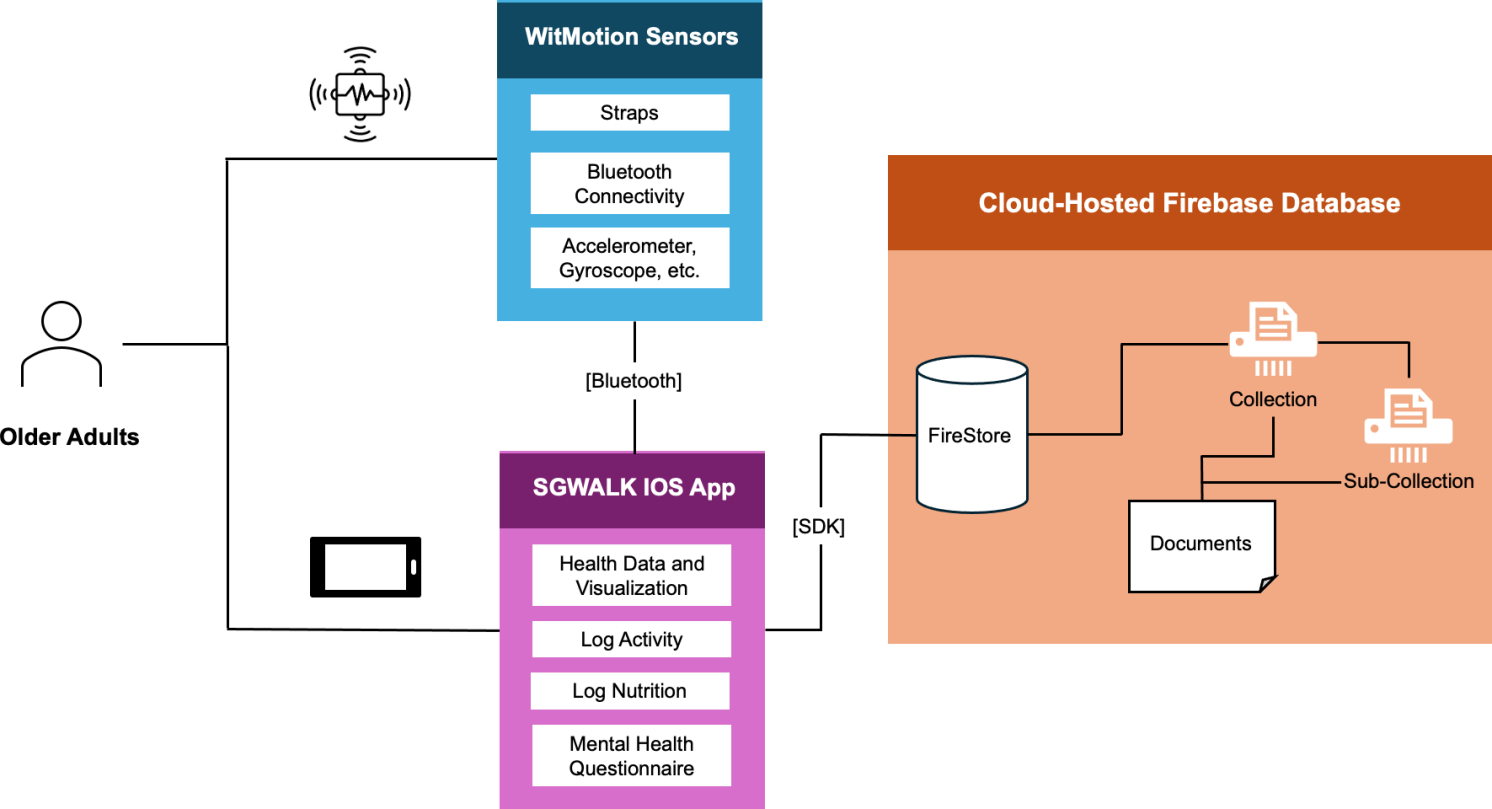
Architecture diagram of SingaporeWALK (SGWALK) app showing data flow between wearables, mobile app, and cloud database. SDK: software development kit.

To address these identified limitations in existing digital health technologies, we developed the SGWALK app using a comprehensive, integrated approach (Figure 1). The architecture of the SGWALK app establishes a connection between wearables and the mobile application via Bluetooth connectivity, thereby establishing a user-friendly platform for monitoring and managing health and mobility. At its essence, the SGWALK app serves as the main interface for end users, offering user-friendly functionalities to access activity data, establish personal goals, and participate in gamified exercises. To effectively capture and monitor users’ physical activity, the SGWALK app interfaces with wearable IMU sensors. These sensors capture movement-related data such as accelerations and angular velocities, facilitating real-time data synchronization between the app and wearables to ensure accurate tracking of the users’ mobility and health metrics. The architecture further incorporates a cloud-hosted Firebase database, serving as the backbone for storing and managing collected data.

#### Main Components and Functionalities

The SGWALK app provides a comprehensive digital platform for older adults to track and manage their health across multiple domains. The system integrates 3 core components: wearable IMU sensors for motion capture, the mobile application interface, and cloud-based data storage.

The app incorporates three Xbox Kinect exergames specifically selected to align with Singapore’s recommended exercises for older adults: (1) Fruit Ninja: players use arm movements to slice digital fruits, promoting upper body mobility and agility; (2) Piano Step: a rhythm-based game requiring synchronized leg movements to hit virtual piano keys, enhancing lower body coordination and balance; and (3) Arctic Punch: players punch ice cube targets to improve arm strength, precision, and coordination. These games were systematically selected based on their movement compatibility with conventional exercises while incorporating gamification elements to ensure sustained participation.

Beyond physical activity tracking, the app captures mental well-being data through standardized questionnaires and nutrition information via user-friendly input interfaces. All data is securely stored in a cloud-hosted Firebase database that enables real-time synchronization and long-term tracking across sessions. The app provides immediate feedback through data visualizations and supports goal-setting features to encourage sustained engagement.

#### User Interface Design Framework

The SGWALK interface was developed using a participatory design methodology involving 3 cycles of iterative testing with 32 older adults (mean age 69.4, SD 6.1 y). Grounded in the Fogg behavior model [16], the design emphasizes 3 key elements: motivation through gamification, ability through accessibility features, and triggers via context-aware prompts. This approach specifically addressed the digital disconnect identified in our needs assessment, where 78% of seniors reported abandoning health apps within 2 weeks due to frustration with complex interfaces.

**Figure 2.**
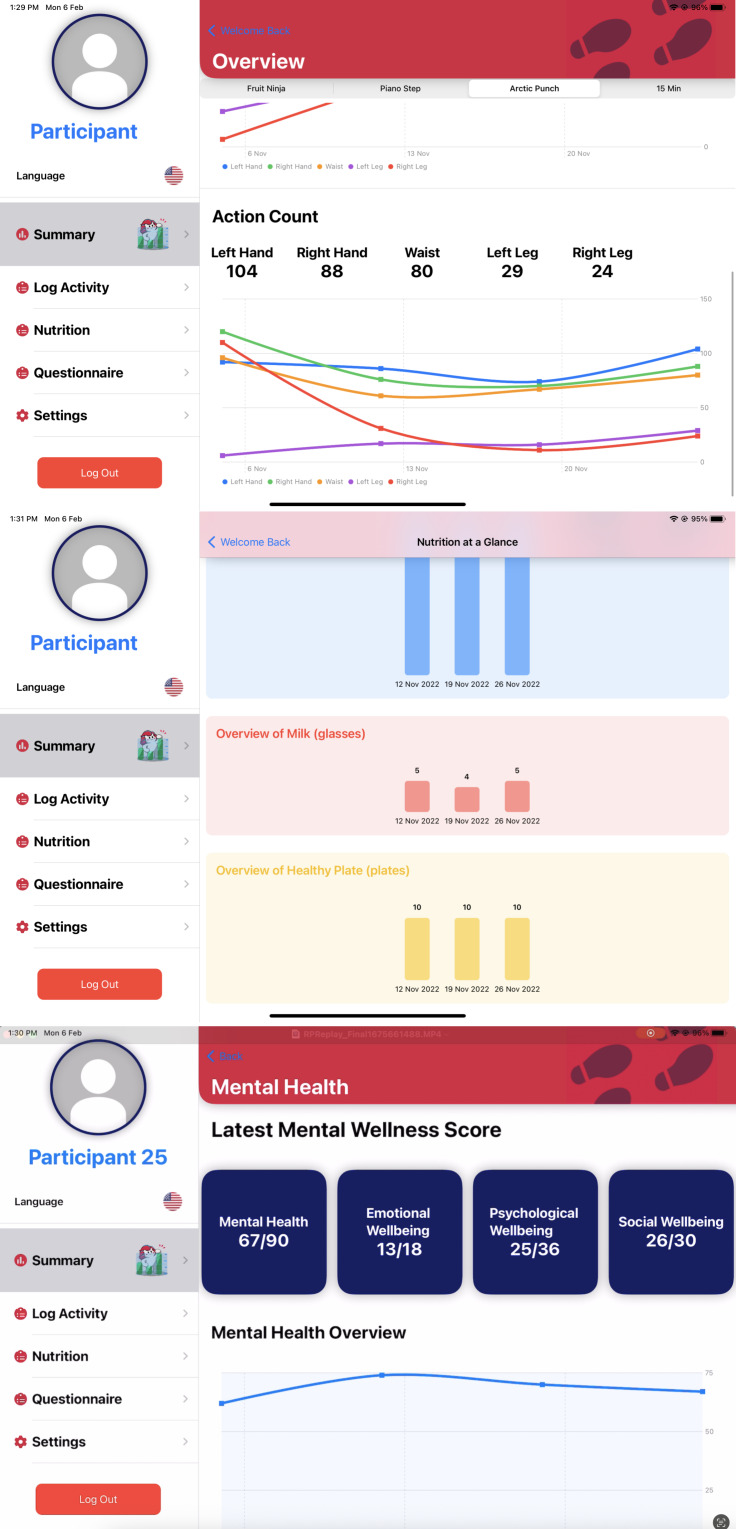
Summary of physical activity metrics (top), nutritional intake (middle), and mental well-being scores based on questionnaire responses (bottom).

The summary tab (Figure 2) presents integrated health metrics using visualizations refined through eye-tracking studies with older adults. Radial progress bars display daily activity goals, demonstrating 87% recognition accuracy versus 52% for numerical displays in usability testing, while sparklines depict weekly trends for mental well-being scores. The nutrition panel uses culturally adapted icons specifically designed for Singapore’s context—a rice bowl for carbohydrates and a fish silhouette for protein—which improved comprehension by 41% compared to standard USDA icons during validation testing.

Navigation follows a strict 3-tap rule validated in gerontechnology literature, with the bottom-aligned menu bar persisting across all screens for consistent accessibility. Tactile feedback was implemented using Apple’s haptic feedback API with medium intensity, a setting preferred by 83% of users in A/B testing. The log activity interface incorporates temporal landmarks (“After breakfast walk”) rather than military time, reducing scheduling errors by 63% in field trials.

The mental health assessment module uses a 14-item adaptation of the Mental Health Continuum–Short Form (MHC-SF) with modified Likert scales using emoji anchors rather than text-only responses. This configuration increased completion rates from 58% to 89% compared to traditional text-only versions, significantly improving user engagement with mental health tracking. For nutrition tracking, the interface implements predictive text entry with Singaporean dish names and portion estimation using familiar hand-size references, specifically addressing the calorie underreporting commonly observed in older adult populations.

Three technical adaptations warrant particular attention for their impact on older adult usability. First, the dynamic text sizing system automatically adjusts layout elements when font sizes exceed 20 pt, preventing interface element overlap that commonly frustrates older adult users. Second, the context-sensitive “Help” button triggers contextual video tutorials demonstrating the current screen’s functionality, providing immediate assistance without navigation complexity. Third, a specialized color correction algorithm adjusts interface hues based on ambient light sensor data to maintain minimum 4.5:1 contrast ratios under varying lighting conditions, ensuring consistent readability across different environments.

## Methods

### Study Design and Participants

This study used a pre-post experimental design to evaluate the effectiveness of the SGWALK app in enhancing physical and mental well-being among older adults. This design was selected for several reasons: it allows for direct comparison of outcome variables before and after the intervention, provides preliminary evidence on usability and effectiveness for innovative technology, and accommodates practical constraints typical in early stages of health technology development [17-19].

Participants were Singaporean citizens or permanent residents aged over 60 years who were physically capable of standing and walking independently and proficient in either English or Chinese. Following written informed consent procedures, 51 participants were randomly allocated to one of four experimental conditions: (1) conventional exercise group, (2) exergames-only group, (3) exergames with health coach support group, or (4) exergames with peer support group. In total, 3 participants discontinued due to personal reasons, yielding a final sample of 48 participants (aged 60‐85 y) who completed the full 4-week intervention protocol. The sample size was determined based on recruitment challenges for older adults in 4-week interventions, the exploratory nature of testing newly developed health technologies, and limited resources for large-scale studies [20,21]. Each participant received an honorarium of S$100 upon study completion.

### Intervention Protocol

The 4-week intervention consisted of weekly 30-minute supervised sessions conducted at community centers with participants allocated to 4 conditions. The conventional exercise group participated in Singapore’s evidence-based 7 Simple Exercises program targeting muscular strength and power enhancement. Exergaming groups used the SGWALK app, which integrates 3 core functionalities. Physical activity promotion was achieved through 3 Xbox Kinect exergames: Fruit Ninja (upper body slicing motions), Piano Step (rhythm-based stepping for lower body engagement), and Arctic Punch (full-body boxing for coordination). These games were selected to align with Singapore’s recommended exercises for older adults. The app featured nutrition tracking with goal-setting for hydration, dairy intake, and balanced meals based on Singapore Health Promotion Board guidelines, and mental well-being assessment using the standardized MHC-SF [14]. Accessibility features included bilingual (English-Chinese) interfaces, enlarged visual elements, and real-time feedback through data visualizations.

Exergaming groups engaged with each game for 10 minutes per session, supervised by trained facilitators. The health coach-supported condition included real-time professional guidance for movement technique correction, performance feedback, motivational support, and safety monitoring. The peer-supported condition introduced collaborative gameplay to foster social connectedness and enhance motivation through peer modeling while maintaining equivalent physical exertion levels across all conditions.

### Data Collection and Measures

Data collection involved multiple assessment methods across the 4-week intervention period. Movement kinematics were continuously monitored using 5 WitMotion IMUs with standardized placement: 2 sensors on the distal forearms (2 cm proximal to the ulnar styloid process), 1 at the L4 vertebral level on the waist, and 2 on the dorsal aspect of both feet. Sensors were secured using medical-grade adjustable straps and hypoallergenic adhesive tape to ensure consistent positioning without restricting natural movement.

Participants completed 20-minute pre- and postintervention questionnaires assessing self-reported physical health, mental well-being, and attitudes toward digital health technologies. Throughout the intervention, participants logged daily nutrition intake and completed weekly mental well-being assessments via the SGWALK app using the MHC-SF. The app provided real-time feedback on physical activity metrics, including action count and maximum acceleration, through line chart visualizations. For analysis purposes, mental health classification divided participants into “mentally well” and “moderate” groups based on MHC-SF scores [14]. Participants were classified as mentally well if they reported experiencing at least 1 of 3 hedonic signs and at least 6 of 11 eudemonic signs “every day” or “5‐6 times a week.”

### Data Analysis

Raw motion data underwent processing to extract meaningful physical activity metrics using established techniques [22-24]. Key measures included:

- *z* score peak detection: This technique identifies significant activity changes by detecting peaks in the accelerometer data using the following formula: *z*=(X - μ)/σ, where X is the data point, μ is the mean, and σ is the standard deviation.
- Action count: It is calculated as the normalized peak count for each game and date range using: Action count=(number of peak counts)/(number of data points)
- Maximum acceleration: The highest acceleration recorded during a specified date range: Maximum Acceleration=max(a), where a represents the acceleration data.
- Root mean square (RMS) acceleration: This provides a measure of overall movement intensity, calculated as: $RMSAcceleration=\sqrt{\frac{1}{N}\sum_{i=1}^{N}a_{i}^{2}}$, where $a_{i}$ represents the acceleration data points and n is the total number of data points.
- Aggregate calorie count calculation: This estimates the total calorie expenditure for each game based on the accelerometer data. It involves calculating the metabolic equivalent of task (MET) from the RMS acceleration using the following formula: METs=1.8×RMS acceleration−15. Energy expenditure (in kcal/h)=1.05×METs×D× W, where D is the duration of activity and W is the participant’s weight.

Statistical analyses used 2-tailed paired *t*-tests to compare pre- and postintervention measures. Physical activity trends were analyzed using temporal comparisons across the 4-week period, with separate analyses for mentally well and moderate participant groups. Nutritional intake patterns and well-being metrics were examined through descriptive trend analysis. All analyses were conducted with a significance level set to identify statistically significant results (*P*<.05).

### Ethical Considerations

Ethical approval was obtained from the Nanyang Technological University Institutional Review Board (IRB-2022‐734). Written informed consent was obtained from all participants after a full briefing on procedures, potential risks, and the right to withdraw without penalty. Participants received compensation of SGD $100 (US $77.73) for their involvement in the study. Data privacy and confidentiality were ensured through deidentification procedures, with participants registering usernames rather than real names in the app. All data were securely stored in the Firebase cloud database for research purposes only.

## Results

During the study period, the SGWALK app provided real-time feedback on participants’ physical activity metrics through the app interface, displaying key metrics such as action count and maximum acceleration. These metrics were presented as line charts for each game, enabling participants to track performance changes over time. Health coaches supplemented this technical data with interpretative guidance to help participants understand their performance data.

**Figure 3.**
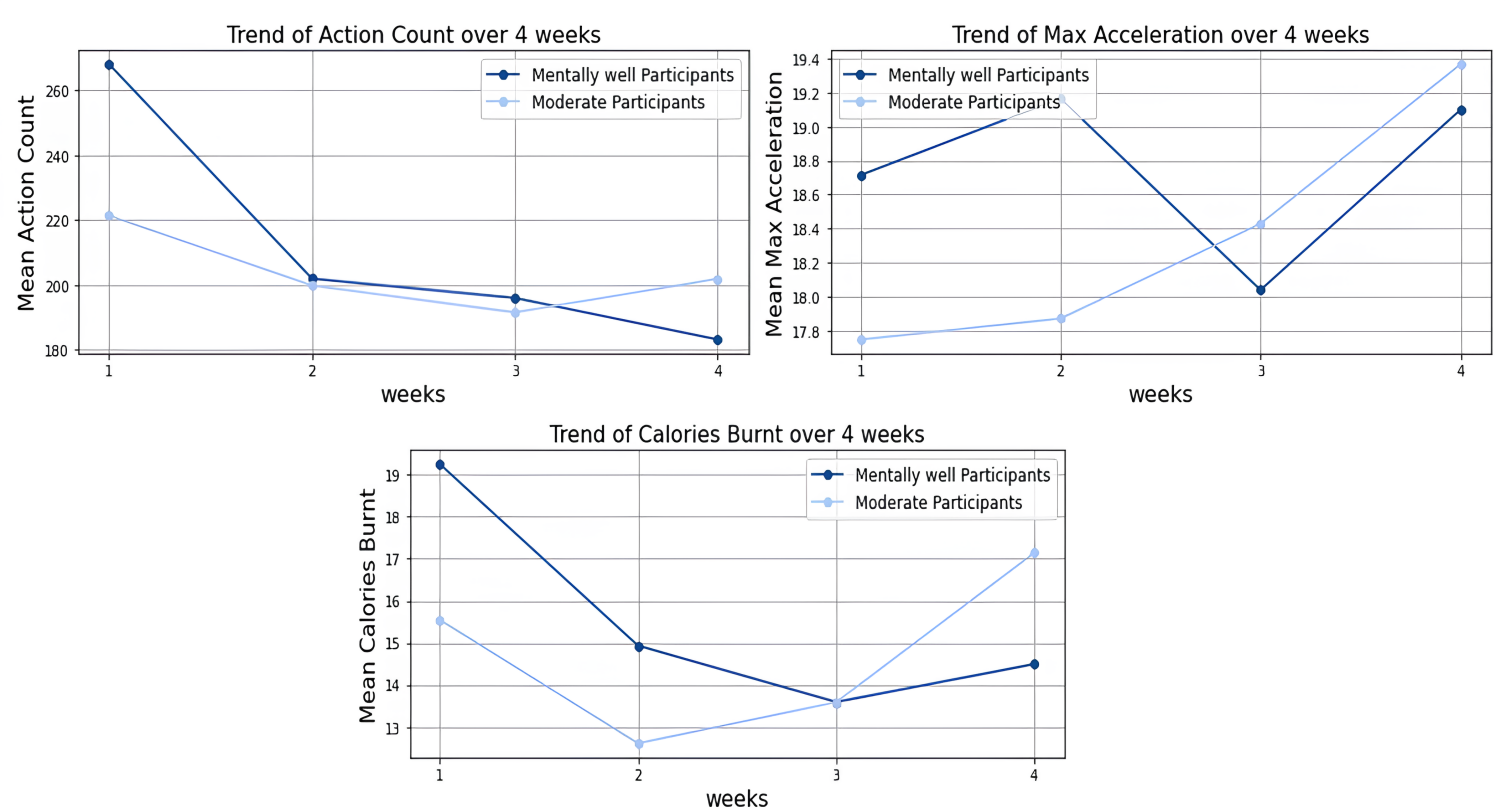
Trend of physical activity metrics over 4 weeks of study.

Postintervention data analysis examined how participants in the defined mental wellness categories (“mentally well” and “moderate”) performed on physical activity metrics. The trend analysis (Figure 3) revealed that mentally well participants consistently demonstrated higher performance compared to the moderate group across most metrics throughout the study period. An important pattern emerged when examining temporal changes: while action count and calories burned gradually decreased over the 4 weeks for both groups, acceleration measurements showed a contrasting pattern with gradual increases over time. This inverse relationship suggests participants may have developed more efficient movement patterns as they became more familiar with the games, enabling them to achieve higher acceleration with fewer discrete actions.

**Figure 4.**
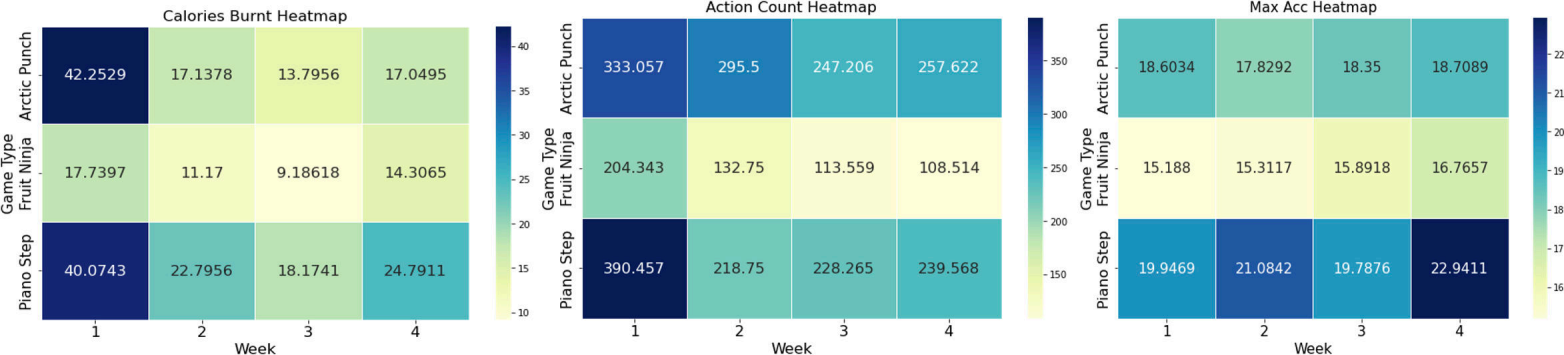
Heatmap of physical activity metrics over 4 weeks of study for all participants.

Physical activity levels showed improvement across the 4-week intervention period. Maximum acceleration increased for both groups, with the mentally well group showing an increase from 18.7 to 19.1 (2.14% improvement) and the moderate group progressing from 17.7 to 19.4 (9.60% improvement), representing an overall 6.5% enhancement (*t*_47_=3.82, *P*<.001). Although the moderate group showed a greater percentage increase, the mentally well participants maintained higher absolute levels throughout the study period. The heatmap analysis (Figure 4) confirmed consistent trends across all participants. While action counts and calories burned decreased over the 4-week period, maximum acceleration values remained stable or slightly increased, supporting the observation of more efficient movement patterns developing over time.

Nutritional intake data demonstrated positive trends throughout the four-week intervention. The analysis revealed that participants maintained a remarkably steady milk intake, indicating successful incorporation of dairy products into their dietary regimen. Additionally, water consumption showed a gradual yet consistent increase, suggesting improved hydration habits as participants progressed through the program. The most significant change was observed in healthy plate intake, which exhibited substantial increases over the four weeks. This upward trend reflects participants' growing commitment to balanced dietary practices and highlights their dedication to nutritional wellness during the intervention. Overall, these findings provide compelling evidence that the SGWALK app intervention effectively facilitated meaningful improvements in participants' dietary habits, potentially fostering patterns that could extend beyond the study period.

**Figure 5.**
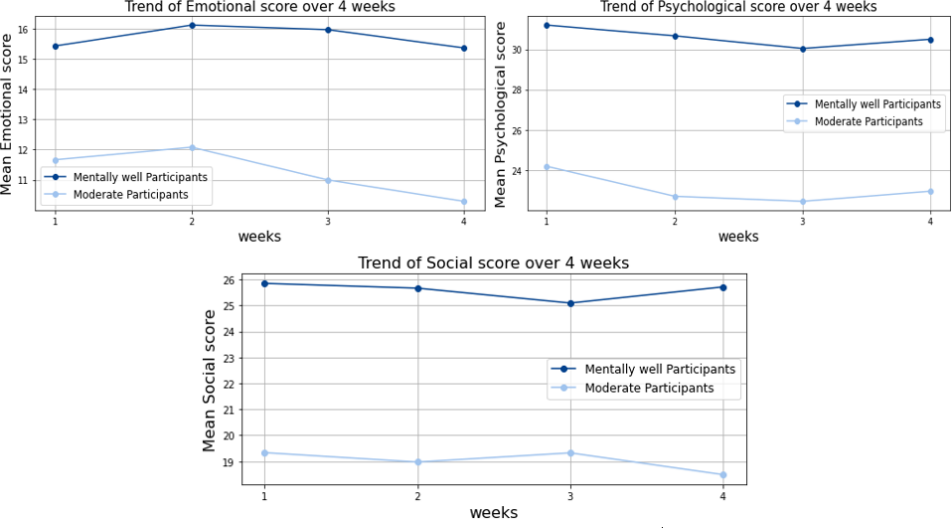


Analysis of emotional, psychological, and social well-being trends (Figure 5) revealed consistent differences between participant groups. Mentally well participants consistently reported higher mean scores across all 3 well-being dimensions compared to moderate participants throughout the intervention period. Emotional well-being scores maintained consistency for both groups throughout the intervention. In contrast, psychological and social well-being scores exhibited greater variability across weeks, particularly among moderate participants, suggesting these dimensions may be more responsive to external influences.

Analysis of *t* tests on questionnaire data revealed significant improvements across 6 of the 7 dimensions measured (Table 1). The most substantial improvements were observed in participants’ perception of ease of use for health apps or wearables (*t*_29_=−4.08, *P*<.001, *d*=1.08) and willingness to use health apps (*t*_29_=−3.63, *P*<.001, *d*=0.96), both representing large effect sizes. Participants’ attitudes toward using health apps or wearables also improved significantly from pre- to postintervention (mean 3.34-3.78, *t*_29_=−2.35, *P*=.02, *d*=0.64). Additional significant improvements with medium effect sizes were found in participants’ perception of how beneficial health technology has been in their lives (*t*_29_=−2.70, *P*=.01, *d*=0.72) and their perceived availability of resources and knowledge about health apps and wearables (*t*_29_=−2.53, *P*=.01, *d*=0.67). Participants also reported significantly better self-rated health over the past week following the intervention (*t*_29_=−2.10, *P*=.04, *d*=0.55). Only one measure showed no significant change: frequency of feeling happy or satisfied with life over the past week (*t*_29_=−1.03, *P*=.31, *d*=0.28). Overall, these findings suggest the SGWALK intervention was effective in enhancing multiple aspects of health technology acceptance and self-perceived health among older adults.

**Table 1.** Paired *t*-test comparison between pre- and postsurvey responses. All tests were 2-tailed paired *t*-tests. Effect sizes (Cohen *d*) calculated using pooled standard deviation.

Item	Survey question	*t* test (*df*)	*P* value	Presurvey, mean (SD)	Postsurvey, mean (SD)	Cohen *d*
Q1	Rating own health over the past week	−2.10 (29)	.04	3.07 (.78)	3.48 (.70)	0.55
Q2	Frequency of feeling happy or satisfied with life over the past week	−1.03 (29)	.31	4.31 (1.05)	4.57 (.79)	0.28
Q3	Willingness to use health apps	−3.63 (29)	<.001	3.18 (.79)	3.95 (.82)	0.96
Q4	Perception of how beneficial health wearables or apps have been in their life	−2.70 (29)	.01	3.34 (.77)	3.86 (.67)	0.72
Q5	Perception of the ease of use of health apps or wearables	−4.08 (29)	<.001	3.01 (.70)	3.76 (.68)	1.08
Q6	Availability of resources or knowledge about health apps and wearables	−2.53 (29)	.01	2.92 (.84)	3.44 (.73)	0.67
Q7	Attitude toward using health apps or wearables	−2.35 (29)	.02	3.34 (.72)	3.78 (.66)	0.64

## Discussion

### Overview

The SGWALK intervention shows encouraging results for promoting active aging and digital health engagement among Singapore’s older adult population. Statistical analyses revealed significant improvements across several domains, including participants’ willingness to use health apps, perceived ease of use of health technologies, and self-reported health status. These findings suggest that carefully designed digital health solutions may help address some of the challenges associated with aging, particularly within Singapore’s multicultural context.

The 4-week intervention was associated with improvements in participants’ attitudes toward health technologies and self-perceived health status. T-test analyses indicated enhanced willingness to engage with health applications and increased perceived ease of use. These changes suggest that structured, supportive interventions may help reduce some older adults’ initial hesitancy toward digital tools, though individual variation in response should be acknowledged. The app’s integration of gamified exercises with visual feedback appeared to contribute to engagement, consistent with previous research demonstrating that gamified elements can enhance motivation in older adult populations [25,26]. The visual-based nutrition tracking features may have supported participants’ reflection on dietary habits, though the mechanisms underlying these changes warrant further investigation [11].

An important finding was the relationship between mental well-being and physical activity performance observed throughout the intervention. Participants classified as “mentally well” consistently outperformed those in the “moderate” category across multiple physical activity indicators, including movement intensity, exercise completion rates, and overall engagement metrics. This pattern suggests a meaningful association between psychological well-being and physical performance capacity among older adults. This relationship aligns with existing literature linking flourishing mental health states to healthy lifestyle behaviors and physical activity engagement [27]. The observed correlation may reflect several underlying mechanisms: individuals with better mental health may possess greater motivation and self-efficacy for physical activities, while conversely, those experiencing moderate mental health challenges may face barriers such as reduced energy, lower confidence, or decreased intrinsic motivation for exercise participation. The bidirectional nature of this relationship is particularly relevant for intervention design. While causality cannot be definitively established from this study design, the findings suggest that mental well-being and physical activity may mutually reinforce each other in older adults. This interplay underscores the importance of holistic approaches when designing digital health interventions, as improvements in one domain may positively influence the other. The improvements in technology acceptance observed are consistent with established theoretical frameworks [28,29], which emphasize perceived usefulness and ease of use as critical determinants of technology adoption. Our results suggest that structured, supportive interventions can shift perceptions even among individuals who may initially be resistant to technology use, reinforcing the value of targeted design and facilitation strategies in overcoming common barriers to digital health tool adoption among older adults.

When compared to existing active aging technologies such as the National Institute on Aging’s Go4Life app [30] and SilverSneakers GO [31], SGWALK offers several distinct advantages. While these existing applications typically focus on isolated health dimensions, primarily exercise routines or fitness programs, SGWALK’s integrated approach simultaneously addresses physical activity, nutrition, and mental well-being assessment. This comprehensive design enables a more holistic understanding of health status and provides users with a unified platform for health management. Several key innovations distinguish the SGWALK intervention from conventional approaches. First, the community-based implementation model goes beyond traditional individual-focused frameworks by embedding digital health engagement within group interaction settings. This strategy not only enhances social connectedness but also leverages peer dynamics to foster engagement and long-term use. Second, SGWALK’s bilingual interface and culturally tailored design elements meet the diverse needs of Singapore’s multicultural older population, ensuring inclusivity and accessibility. Third, by integrating multiple health domains, the app allows for cross-dimensional analyses, for example, how mental well-being relates to physical performance. Lastly, the use of IMU sensors in conjunction with exergames enables more precise and comprehensive tracking of movement compared to conventional step counters, offering richer insights into exercise quality and intensity.

Beyond the anticipated outcomes, the study revealed several unexpected yet important observations. While action counts and calories burned decreased over time, acceleration metrics increased, suggesting participants may have developed more efficient movement patterns as they became accustomed to the activities. This trend offers an interesting direction for future research into motor learning and exercise efficiency among older adults. Implementing SGWALK in community centers also highlighted the importance of social context in technology adoption. Participants showed higher engagement during group sessions, likely influenced by familiar environments and social interaction. Although these observations were qualitative, they point to the potential benefits of socially anchored digital interventions. The significant improvements in technology acceptance further support the idea that trusted, supportive environments, when paired with thoughtful exposure, can help overcome common barriers to adoption among older adults.

In total, 2 key design lessons emerged from the implementation process. First, striking the right balance between technological sophistication and ease of use is critical. While features like real-time feedback and activity tracking enhanced engagement for some, others found overly complex navigation or cognitive load challenging. Simplified interfaces, intuitive icons, and clear visual cues proved instrumental in facilitating uptake. Second, embedding the app within Singapore’s established network of older adult activity centers was pivotal in supporting sustained participation. These familiar, supportive settings reduced apprehension, fostered peer motivation, and encouraged continuous engagement.

### Limitations and Future Directions

While the SGWALK intervention shows promising results, several limitations should be acknowledged. The sample size of 48 participants, though sufficient for initial evaluation, may not fully represent the diversity of Singapore’s older adult population. Future research should prioritize larger-scale studies with more representative sampling. The relatively brief 4-week intervention duration limits our understanding of long-term engagement patterns and sustained impact. Longitudinal studies spanning several months would provide valuable insights into the temporal evolution of usage patterns and determine whether observed benefits persist beyond the initial engagement period. Future development of the SGWALK platform should focus on several key areas. Enhancing cross-cultural adaptability would extend the app’s applicability to different cultural contexts. Implementing personalization algorithms could tailor content based on individual user characteristics and preferences. Exploring integration with additional health monitoring technologies could expand the platform’s health management capabilities. To support user engagement, future iterations should consider implementing additional gamification elements, more personalized feedback mechanisms, and features that enable social interaction within the app. As user numbers grow, robust data management infrastructure will become increasingly important to ensure data security while maintaining user privacy.

### Conclusions

The SGWALK app represents a significant advancement in digital health interventions for older adults, offering a culturally responsive, holistic approach to promoting active aging through accessible technology. Our findings provide practical frameworks for adapting health technologies to multicultural older adult populations, implementing wearable sensor technology in community settings, and integrating physical activity tracking with mental well-being assessment. As global populations continue to age, interventions that successfully combine technological innovation with cultural sensitivity will become increasingly important. Digital health solutions that prioritize both effectiveness and accessibility can contribute to enhancing quality of life and promoting independence among older adults worldwide.

## Abbreviations

IMU: inertial measurement unit
MET: metabolic equivalent of task
MHC-SF: Mental Health Continuum–Short Form
mHealth: mobile health
RMS: root mean square
SGWALK: SingaporeWALK (Wearables and Apps for community Living and Knowledge)
